# A Textbook of Pharmacology and Therapeutics

**Published:** 1910-12

**Authors:** 


**A Textbook of Pharmacology and Therapeutics.** By **Arthur R. Cushny** M.A., M.D., F.R.S., Professor of Pharmacology in the University of London. Fifth edition. Octavo, 774 pages, with 61 engravings. Lea & Febiger, Publishers, Philadelphia and New York. 1910. (Cloth, $3.75, net.)

To meet the everchanging nature of an unfinished science necessitating alterations in a number of chapters in this textbook, the opportunity has been taken to revise the whole work thoroughly.

The amount and importance of the researches published during the past few years testify to the growing interest in the action of drugs and their application.

Important advances have been made in the study of individual drugs which permit of a more definite statement of their action. The author has taken advantage of this addition to our knowledge in his new revision. He states the tendency of the times seems to be to control rather than to increase remedial agents.

